# Characterization of an Acetogenin-Carrying Nanosuspension and Its Effect on Bacteria of Interest in the Poultry Industry

**DOI:** 10.3390/microorganisms13010018

**Published:** 2024-12-25

**Authors:** Brandon A. López-Romero, Gabriela Aguilar-Hernández, Billy M. Hargis, María de Lourdes García-Magaña, Ulises M. López-García, Rosa I. Ortiz-Basurto, Adalberto Zamudio-Ojeda, Juan D. Latorre, Efigenia Montalvo-González

**Affiliations:** 1 Laboratorio Integral de Investigación en Alimentos, Tecnológico Nacional de México, Instituto Tecnológico de Tepic, Tepic 63175, Mexico; brallopezro@ittepic.edu.mx (B.A.L.-R.); mgarciam@ittepic.edu.mx (M.d.L.G.-M.); ulopez@ittepic.edu.mx (U.M.L.-G.); riobasurt@ittepic.edu.mx (R.I.O.-B.); 2 División de Ciencias Agropecuarias e Ingenierías, Centro Universitario de los Altos, Universidad de Guadalajara, Guadalajara 47600, Mexico; gaby.mca2017@gmail.com; 3 Department of Poultry Science, University of Arkansas, Fayetteville, AR 72701, USA; bhargis@uark.edu; 4 Departamento de Física y Química, Centro Universitario de Ciencias Exactas e Ingenierías, Universidad de Guadalajara, Guadalajara 44430, Mexico; adalberto.zojeda@academicos.udg.mx

**Keywords:** acetogenins, antibacterial activity, nanosuspensions, poultry sector

## Abstract

This work aimed to develop a nanosuspension (NSps) as an acetogenin (ACGs) carrier, using soy lecithin (SL) and hydroxypropyl-β-cyclodextrin (βCD) named NSps-βCDSL-ACGs. It was characterized by various spectroscopic techniques (DLS, FTIR, UV-vis diffuse reflectance). Moreover, the NSps morphology was observed by transmission electron microscopy (TEM). Also, the antibacterial activity of NSps-βCDSL-ACGs was evaluated against strains of interest in the poultry sector. NSps-βCDSL-ACGs presented nanometric size (207–239 nm), acceptable polydispersity index (PDI) values (0.13–0.17) and a high Z potential value (−47.17–50.36 mV), demonstrating high stability. The presence of ACGs in NSps-βCDSL-ACGs was confirmed by FTIR analysis. The nanoparticles had a spherical shape and exhibited high inhibition potential against *Salmonella* Enteritidis (88.18%), *Streptococcus gallolyticus* (88.01%), *Salmonella* Typhimurium (86.28%) and *Salmonella* Infantis (77.02%) strains up to 48 h, and a reduction of up to 3 log CFU/mL was achieved for *S.* Typhimurium. Therefore, NSps-βCDSL-ACGs is an attractive option for implementing ACGs administration in the poultry sector to reduce the use of antibiotics and minimize bacterial resistance.

## 1. Introduction

**Figure 1:**
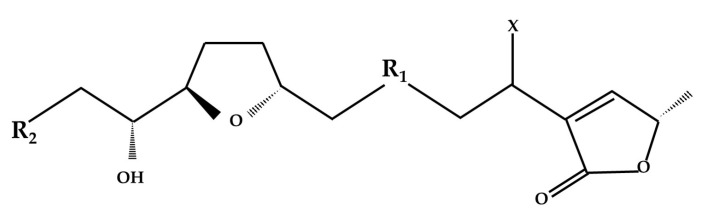
The general structure of an Annonaceous acetogenin. X is the OH or Oxygen group, R_1_ and R_2_ are carbon radicals.

Acetogenins (ACGs) are bioactive compounds extracted from different parts of Annonaceae plants. They are characterized by having a long aliphatic chain with 35 to 37 carbon atoms, 1-3 tetrahydrofuran or tetrahydropyran rings in their central region, an unsaturated or saturated γ-lactone-α-β and OH, or Oxygen groups in any part of their chain, as shown in Figure 1 [1,2].

ACGs are of pharmaceutical interest because they have been used effectively as antitumor, cytotoxic and antimicrobial compounds, which is attributable to the fact that they inhibit NADH ubiquinone oxidoreductase of mitochondrial complex I, which causes a reduction in ATP synthesis and consequently trigger programmed cell death [1,3,4]. However, despite their potential, the application of ACGs in various in vitro and in vivo activities is limited due to their hydrophobic character, so in some studies, it has been necessary to incorporate them in nanocarrier systems that help to improve their administration and efficacy, as is the case of nanosuspensions (NSps) [5,6,7,8,9,10]. NSps are colloidal systems that use mechanical energies and the incorporation of polymers and surfactants to decrease the particle size and stabilize hydrophobic compounds in aqueous media, thus increasing the efficacy of the encapsulated compounds [11]. Therefore, recent studies have evaluated the antiproliferative and antimicrobial effects of NSps as carriers of ACGs. In this regard, López-Romero et al. [9] and Montalvo-González et al. [10] evaluated the antibacterial effect of NSps loaded with ACGs (320 µg/mL) using β-cyclodextrin (0.16% *w*/*v*) and polyethylene glycol 6000 (0.2% *w*/*v*) as wall polymer and soy lecithin (10–15 mg/mL) as surfactant. The results showed that NSps with β-cyclodextrin and PEG showed high inhibition against *Enterococcus faecalis* (83–87%) and *Listeria monocytogenes* (75–86%), respectively. In contrast, Aguilar-Hernández et al. [12] evaluated high concentrations of isolated ACGs (2000 and 4000 µg/mL) for only 30% inhibition of *E. faecalis* and up to 92% inhibition of *L. monocytogenes*, demonstrating the potential of NSps as an ACGs carrier, in order to increase their effect and efficacy.

On the other hand, in recent years, the poultry industry has seen an increase in cases of bacteria-related diseases that significantly affect this sector, causing significant economic losses [13]. Intensive animal production and antimicrobial resistance have led to a rise in bacterial diseases, especially diseases attributed to food-borne pathogens of the genus *Salmonella* [14]. Antibiotics have been the primary method of controlling bacterial diseases in livestock; however, the effectiveness of this control method has decreased due to the emergence of antibiotic-resistant strains [15]. The indiscriminate use of antibiotics can cause genetic changes and lead to the emergence of resistant bacteria. As a result, 78% of the regions of Africa and South America are expected to have bacteria resistant to tetracyclines and penicillin within 1.7 to 12.4 years [16].

In recent years, there have been reports on the prevalence of *Salmonella* Enteritidis (68.1%) and *Salmonella* Typhimurium (31.8%) in broiler chickens. These serotypes showed resistance to various antibiotics, including polymyxin-B (81. 8%), nalidixic acid (77.27%), colistin (59.1%), ampicillin/tetracycline (45.5%), ampicillin + sulbactam (40.9%), cefadroxil (18.2%), streptomycin (9.1%), and ceftazidime/ceftriaxone-tazobactam (4.5%) [17]. Due to the risk posed by the emergence of strains resistant to different antibiotics, the poultry industry has had to find alternatives for the control of bacteria present in food animals. Among these is the use of natural extracts from garlic, fennel, oregano and chamomile, which are made up of various bioactive compounds with the antimicrobial effect that help promote the immunological activity of the animal. However, the results obtained have not been sufficient to consistently control the bacteria that harm this sector [18,19,20,21].

Therefore, examining the possible impact of nanosuspensions as sources of acetogenin (NSps-ACGs) against problematic highly pathogenic bacteria affecting the poultry industry is interesting. At the same time, providing an alternative could contribute to decreasing the use of antibiotics and thus minimizing the risk of antimicrobial resistance in the future. In our laboratory, two types of NSps-ACGs have been developed from different stabilizers; however, in this work, we used the NSps previously developed by López-Romero et al. [9] because it showed the most significant antimicrobial effect. In this experiment, the ACGs concentration in NSPs was changed; therefore, the new NSps had to be characterized, and their antibacterial activity was evaluated against four antibiotic-resistant bacterial strains, which are most prevalent in chickens and chicken embryos.

## 2. Materials and Methods

### 2.1. Materials

The ACGs used in this study were extracted from *Annona muricata* seeds by thermosonication, and the extract was purified by column chromatography and characterized by HPLC, where 10 acetogenins were found to be present, with pseudoannonacin (60%) being the most critical [22]. Ethyl alcohol (96°), (2)-hydroxypropyl-β-cyclodextrin (βCD), and soy lecithin (SL) were acquired from Sigma Aldrich (St. Louis, MO, USA). Tryptic soy broth (TSB), tryptic soy agar (TSA), brain and heart infusion broth (BHI), and Columbia agar (CNA) with nalidixic acid and colistin with 5% ram blood were purchased from Beckton Dickinson and Company (Le Pont de Claix, France).

### 2.2. Bacterial Strains

The John Kirkpatric Skeeles Poultry Health Laboratory of the University of Arkansas provided *Salmonella* Enteritidis, *Salmonella* Typhimurium, *Salmonella* Infantis, and *Streptococcus gallolyticus* strains. The strains were handled according to the specifications reported for each strain. All materials and reagents used for this determination were previously sterilized for 15 min, 17 psi, and 121 °C.

### 2.3. Preparation of Acetogenin-Loaded Nanosuspensions

NSps loaded with ACGs were developed using the López Romero et al. [9] method with some modifications. Briefly, βCD (0.16% *w*/*v*) dissolved in distilled water (10 mL) and 800 µL of a soy lecithin solution (SL; 10 mg/mL ethanol) was added dropwise and stirred at 500 rpm for 30 min until the amphiphilic complex (βCDSL complex) was obtained.

Ethanol was evaporated from the βCDSL complex using a rotary evaporator (Yamato RE300, Tokyo, Japan). Subsequently, 160, 480, and 800 µL of ACGs [62.5 mg/mL in ethanol] were added dropwise and shaken (500 rpm) for 30 min. Finally, ethanol was removed, and distilled water was added to achieve ACGs-loaded NSps (NSps-βCDSL-ACGs) 1, 3, and 5 mg/mL concentrations. βCD and SL are two species that self-assemble into an amphiphilic complex due to the polar groups of βCD and the phosphate group of SL. Then, the SL (initially bound to βCD) interacts with its non-polar tail with the ACGs. The βCD on its polar outer layer interacts with water, causing the formed nanoparticle to be in an aqueous medium [5].

### 2.4. Particle Size, Polydispersity Index, and Zeta Potential of NSps-βCDSL-ACGs

Particle size, polydispersity index (PDI), and Z-potential of NSps-βCDSL-ACGs were analyzed in all samples via classical dynamic light scattering equipment at 90° (ZN90, Malvern Instruments, Cambridge, UK). Three replications of NSps analysis were performed at 25 °C.

### 2.5. Spectroscopic Characterization of NSps-βCDSL-ACGs

#### 2.5.1. Fourier Transform Infrared Spectroscopy (FTIR)

NSps-βCDSL-ACGs (5 mg/mL), isolated ACGs (5 mg/mL), βCD (0.16%), SL (10 mg/mL), and the βCDSL complex were analyzed by FTIR (iS10 FTIR, Thermo Scientific, Waltham, MA, USA). Spectra were measured in the 4000 to 500 cm^−1^ range with 24 scans, 4 cm^−1^ resolution, and at room temperature (25 °C).

#### 2.5.2. Diffuse Reflectance UV-VIS Spectroscopy

The NSps-βCDSL-ACGs (5 mg/mL), the βCDSL complex, and the isolated ACGs were all examined via diffuse reflectance spectroscopy using a UV-Vis spectrometer (Shimadzu UV-2600, Tokyo, Japan) supplemented with an integrated sphere for diffuse reflectance studies. The UV-VIS absorption spectrum ranged from 190 to 600 nm within the spectral spectrum.

#### 2.5.3. Transmission Electron Microscopy (TEM)

TEM analysis was performed to examine the nanoparticle size of NSps-βCDSL-ACGs (5 mg/mL). The sample was placed on a copper support grid and allowed to dry. Images were then captured using a JEM-1010 transmission microscope (JEOL, Tokyo, Japan) at 60 Kv [23].

### 2.6. Determination of the Antimicrobial Activity of NSps-βCDSL-ACGs

The antibacterial effect of NSps-βCDSL-ACGs was evaluated using the microdilution method reported by Montalvo-González et al. [10] with some modifications. TSB or BHI (100 μL) was added to each well, 125 μL of NSps-βCDSL-ACGs at 1, 3, and 5 mg/mL to reach a final concentration of 0.5, 1.5, and 2.5 mg/mL of ACGs, βCDSL complex (amphiphilic complex without ACGs), negative control (TSB or BHI with bacteria), or antibiotic control using the reported minimum inhibitory concentration (MIC) of ampicillin for *S.* Enteritidis (2.5 μg/mL), *S.* Typhimurium (0.75 μg/mL), *S.* Infantis (2 μg/mL), and *S. gallolyticus* (0.5 μg/mL) [24,25]. Each well was inoculated with 25 μL of a bacterial suspension at 1 × 10^6^ CFU/mL. The sample was incubated at 37 °C, and the absorbance (545 nm) was recorded every hour for the first 6 h, continuing every 6 h until 48 h of incubation. The percentage of bacterial inhibition (BI) was determined by Equation (1).

(1) $$BI(\%)=\frac{ANC-ANS}{ANS}\times 100,$$

where ANC is the absorbance of the negative control (media with bacteria), and ANS is the absorbance of the NSps-βCDSL-ACGs, βCDSL complex or positive control (antibiotic).

#### 2.6.1. Determination of the Mean Inhibitory Concentration (IC_50_) and Minimum Inhibitory Concentration (MIC) of NSps-βCDSL-ACGs Using a Regression Analysis

With the data obtained in Section 2.6, the IC_50_ for each bacterium was calculated using a linear regression model (sigmoid dose–response with variable slope, R^2^ > 0.99) with the software Graph Pad Prism 8.0 (San Diego, California, EEUU., 2018) [26]. For the calculation of the MIC in each bacterium, the methodology described by Bloomfield et al. [27] was used, which establishes that the concentrations of NSps-βCDSL-ACGs (0.5, 1.5 and 2.5 mg/mL) used to evaluate the antibacterial effect (Section 2.6) are converted to the natural logarithm (ln) and the value obtained is plotted on the X-axis. In contrast, the squared inhibition percentage (%BI^2^) values are represented on the Y-axis. Equation (2) was obtained through a linear regression model, which was used to obtain Equation (3), where “X” is the Ln of the concentration, “y” is the minimum value of %BI^2^, “b” is the intersection of the X-axis, and “m” is the slope. Finally, Equation (4) was used to determine the MIC value of each bacterium studied.

(2) Y=mx+b,

(3) $$X=\frac{y-b}{m},$$

(4) $$MIC=e^{x}\times 0.25.$$

#### 2.6.2. Lethality of NSps-βCDSL-ACGs Against *S.* Enteritidis, *S.* Typhimurium, *S.* Infantis, and *S. gallolyticus*

Lethality was assessed using a serial dilution assay [12]. Mixtures of 25 μL of bacterial suspension (1 × 10^6^ CFU/mL), 100 μL of TSB or BHI, and 125 μL of NSps-βCDSL-ACGs (1, 3 and 5 mg/mL), complex-βCDSL, positive control (ampicillin MIC) or negative control were incubated at 37 °C for 48 h. Subsequently, serial dilutions (up to 10^7^) were performed, and 10 μL of each dilution was semi-diluted on TSA for *S.* Enteritidis, *S.* Typhimurium, *S.* Infantis, or CNA for *S. gallolyticus*. This process was evaluated for each bacterial strain, and the results were expressed as log CFU/mL. Lethality was determined by the difference between the log CFU/mL of the colonies counted in the plates with bacteria treated with the nanosuspensions at different concentrations (TB) and the colonies counted in plates with untreated bacteria (NTB) (Equation (5)):

(5) $$Lethality[LogCFU/mL]=Log\left[NTB-TB\right]$$

### 2.7. Statistical Analysis

All assays were performed in triplicate for each experiment, and the results were reported as the mean ± standard deviation. Data were analyzed by ANOVA and Fisher’s LSD test (*p* < 0.05) using Statistica statistical software (v.10 StatSoft, Tulsa, OK, USA).

## 3. Results and Discussion

### 3.1. Particle Size, Polydispersity Index, and Z-Potential of NSps-βCDSL-ACGs

The corresponding values for particle size, PDI and Z-potential are presented in Table 1. Significant statistical differences (*p* < 0.05) were observed between the different concentrations (1, 3, and 5 mg/mL) of NSps-βCDSL-ACGs. The values corresponding to the particle size were within the range of 207–239 nm, observing that the largest particle size was presented in the NSps prepared with 5 mg/mL of ACGs (239.12 nm), while the smallest size was obtained with the NSps at 1 mg/mL of ACGs (207.34 nm). The difference between the different nanometer sizes of the particles may be due to a large number of qualitative and quantitative factors that influence the size, such as the nature of the encapsulated compound, viscosity, interfacial tension between the phases, and hydrophobicity. However, one of the most important factors is the concentration of the encapsulated drug [28]. In this sense, it can be certified that the variation in particle size was due to the different concentrations of ACGs in the NSps-βCDSL-ACGs. Although the particle size in NSps-βCDSL-ACGs was around 207–239 nm, the sizes found were within the nanometric range for colloidal systems according to the literature (<700 nm), which allows for a higher dissolution rate due to the increase in surface area. In addition, better stability is obtained, and particle aggregation (Ostwald ripening) is avoided [29].

Also, the PDI values in NSps-βCDSL-ACGs (0.13–0.17) present statistically significant differences (*p* > 0.05) between each NSps. However, all the values demonstrate polydispersity in the samples, indicating a uniform particle size population [30]. PDI values can vary from 0.0 for a perfectly uniform sample in particle size to 1.0 for an utterly polydisperse sample with multiple particle size populations. However, particle sizes <0.3 are considered acceptable uniform values [30]. Therefore, the PDI values found in this study are satisfactory, added to a high Z potential value (>30 mV) that demonstrates a high stability of the NSps due to the high electrostatic repulsion forces that prevent the molecular aggregation caused by the collisions of adjacent particles. The Z-potential indicates the surface charge of the particles, which is mainly affected by the dissociation of functional groups through the adsorption of ionic species in the dispersion medium (water) and the solvation effect [28].

### 3.2. Spectroscopic Characterization of Isolated ACGs, Complex βCDSL, and NSps-βCDSL-ACGs

#### 3.2.1. Fourier Transform Infrared Spectroscopy (FTIR)

**Figure 2:**
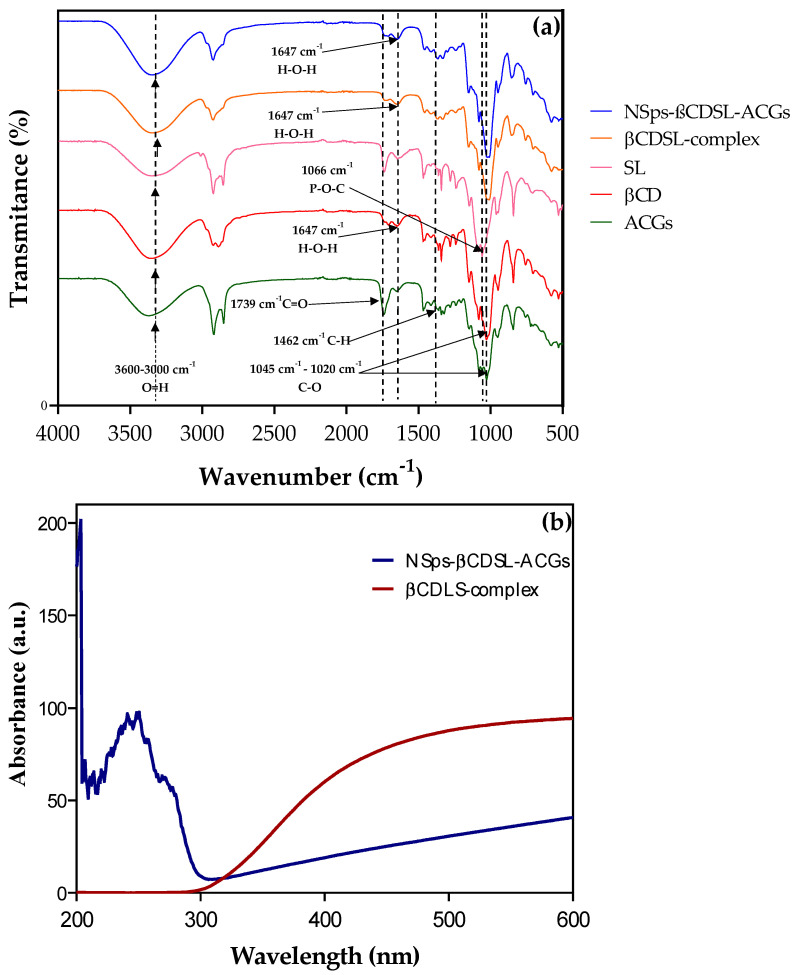
(**a**) Fourier transform infrared spectroscopy (FTIR) of isolated acetogenins (ACGs), hydroxypropyl-β-cyclodextrin (βCD), soy lecithin (SL), complex hydroxy-propyl-β-cyclodextrin with soy lecithin (βCDLS-complex), and NSps-βCDSL-ACGs. (**b**) Diffuse reflectance spectroscopy analysis–UV-VIS of NSps-βCDSL-ACGs and NSps-βCDSL-complex.

FTIR spectra (4000–400 cm^−1^) for NSps-βCDSL-ACGs, βCDSL complex, SL, βCD, and ACGs are presented in Figure 2a. All samples exhibited narrow vibrations in the 3600–3000 cm^−1^ region, corresponding to the O-H groups [9]. The signals observed around 1739 cm^−1^ are assigned to the C-O, indicating the probable presence of the lactone ring characteristic of ACGs; likewise, the axial deformation of the carbon–hydrogen bond (C-H) indicates the portion connected to the tetrahydrofuran ring can be observed in the 1462 cm^−1^ region [31,32,33]. The ACGs and βCD showed peaks in the 1020–1045 cm^−1^ region, corresponding to the carbon–oxygen (C-O) bond. The NSps-βCDSL-ACGs and βCDSL complex show peaks in this region, however, these are broader. The broad peaks are related to the host: host interaction phenomenon that occurs in the formation of inclusion complexes and indicates good compound encapsulation [34]. On the other hand, βCD, βCDSL complex, and NSps-βCDSL-ACGs presented bands in the 1647 cm^−1^ region corresponding to H-O-H bending, while SL presented a peak in the 1066 cm^−1^ region corresponding to the phosphodiester bond (P-O-C) [35]. All the absorption bands of ACGs and SL showed an overlap or were covered by the βCD bands in the βCDSL complex and NSps-βCDSL-ACGs. This behavior has been reported previously for this inclusion complex and indicates that both ACGs and SL entered the βCD cavity and the inclusion complex was successfully formed [35]. The absence of new bands in the spectra of NSps-βCDSL-ACGs indicates that no new chemical bonds were developed. Therefore, the interaction occurred mainly through hydrogen bonds and Van der Waals forces [34]. The FTIR spectra obtained in this study agreed with those previously reported by López-Romero et al. [9] and Montalvo-González et al. [10], where the structure of the ACGs in the NSps was preserved.

#### 3.2.2. Diffuse Reflectance Spectroscopy–UV-VIS Analysis

In Figure 2b, the highest absorbance of the NSps occurs around 200 nm, which is consistent with that reported by López Romero et al. [9], who indicate that this is related to the π-π* transitions due to C=C located in the lactone ring of the ACGs. From this, it follows that when encapsulating acetogenins in the amphiphilic complex, some of the molecules are partially exposed, leading to the detection of absorbance signals in the UV-Vis analysis. On the other hand, it can be seen in Figure 2b that the amphiphilic complex does not show absorption bands up to 600 nm.

This behavior is linked to beta-cyclodextrin, which in its structure does not show absorbance in the UV-vis analysis since it does not have double bonds. However, it has been reported that soy lecithin, also present in the amphiphilic complex, should present absorption bands of around 200 nm due to specific functional groups and double bonds. However, it is essential to note that when an inclusion complex is created between lecithin and beta-cyclodextrin, lecithin may be fully incorporated into the cavity of beta-cyclodextrin. This process causes the disappearance of the absorbance maximum. This results in the disappearance of the absorbance peak of lecithin in the UV-Vis spectrum. Since it is encapsulated in the structure of beta-cyclodextrin, the groups responsible for absorbance are no longer accessible to UV radiation. This behavior suggests that the interaction between the two compounds is effective, and lecithin is inside the inclusion complex [35].

#### 3.2.3. Transmission Electron Microscopy (TEM)

**Figure 3:**
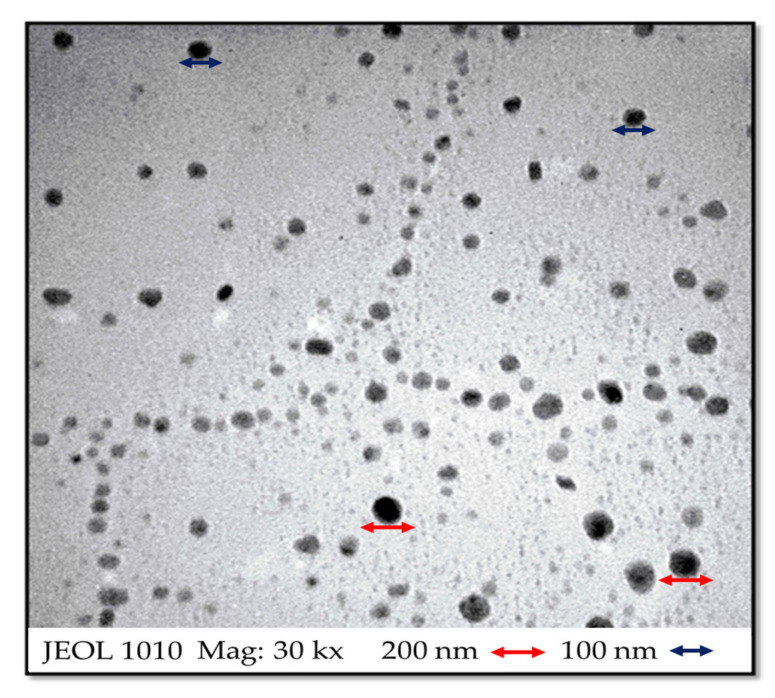
Transmission electron microscopy (TEM) of NSps-βCDSL-ACGs.

TEM analysis (Figure 3) showed that the nanoparticles (100–200 nm) in NSps-βCDSL-ACGs had spherical shapes. TEM micrographs showed that the nanoparticles were in a similar nanosized range to those found by Dynamic light scattering (DLS) (207–239 nm).

### 3.3. Determination of the Antimicrobial Activity of NSps-βCDSL-ACGs

#### 3.3.1. Microdilution Plate for the Determination of Bacterial Inhibition of NSps-βCDSL-ACGs

**Figure 4:**
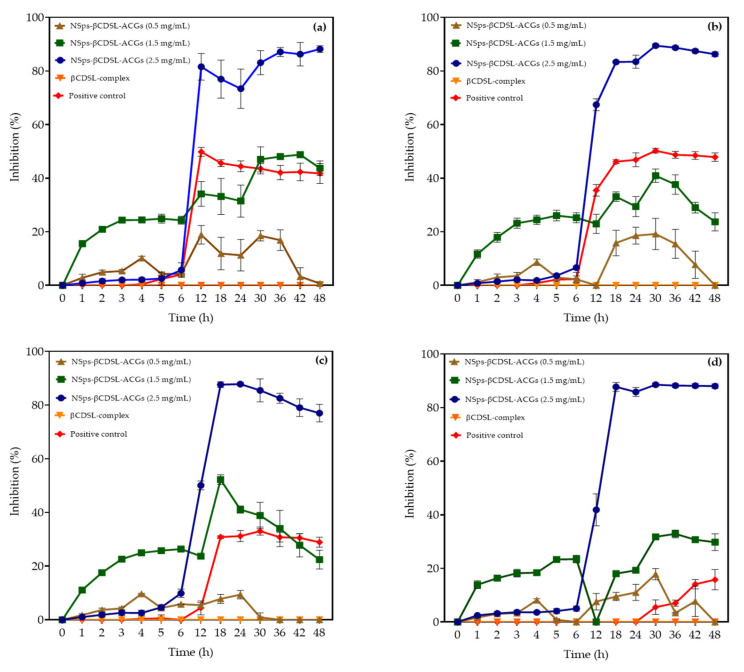
Inhibition of NSps-βCDSL-ACGs against *Salmonella* Enteritidis (**a**), *Salmonella* Typhimurium (**b**), *Salmonella* Infantis (**c**), and *Streptococcus gallolyticus* (**d**). Positive control = ampicillin for *S.* Enteritidis (2.5 μg/mL), *S.* Typhimurium (0.75 μg/mL), *S.* Infantis (2 μg/mL), and *S. gallolyticus* (0.5 μg/mL).

The antibacterial effect of NSps-βCDSL-ACGs against *S.* Enteritidis, *S.* Typhimurium, *S.* Infantis, and *S. gallolyticus* can be observed in Figure 4a–d. These figures show that the antibacterial effect was concentration-dependent, with the 2.5 mg/mL NSps showing the highest bacterial inhibition (77.02–88.01%), while the 1.5 mg/mL of ACGs in NSps reached lower inhibition values (22.43–43.73%) in all strains evaluated. These results were corroborated by the null inhibition presented by βCDSL-complex up to 48 h of exposure. The inhibition depended on each bacterium and the concentration of ACGs in the NSps. The antibacterial activity of ACGs is related to the number of unsaturations in their aliphatic chain because an insertion into the cell membrane occurs. These unsaturations significantly influence their coupling and functionality in relation to the NADH oxidase located in the plasma membrane of bacteria [36]. In addition, ACGs could have a chelating effect, which induces the opening and formation of new pores in the membrane, modifying solubility and intracellular permeability [37].

On the other hand, the bacterial inhibition in this assay was carried out in the following order: *S.* Enteritidis (88.18%) > *S. gallolyticus* (88.01%) > *S.* Typhimurium (86.28%) > *S.* Infantis (77.02%). Moreover, it was observed that the inhibition of the NSps-βCDSL-ACGs at 2.5 mg/mL was higher than the positive control in all the strains evaluated (15.79–47.86%) up to 48 h of exposure.

The different effects of NSps-βCDSL-ACGs for each bacterium are attributed to the fact that the strains evaluated in this work present multiple resistance to antibiotics as a consequence of the modification of specific membrane porins (OmpF, OmpC, or OmpE). These modified porins limit the permeability of the membrane, making the diffusion of drugs into the bacterium difficult. The main factor of porin mutation is the frequent use of antibiotics against bacterial pathogens in chickens and turkeys [38].

#### 3.3.2. Determination of the Mean Inhibitory Concentration (IC_50_) and Minimum Inhibitory Concentration (MIC) of NSps-βCDSL-ACGs Using Regression Analysis

The data corresponding to the IC_50_ and MIC of the NSps-βCDSL-ACGs for each bacterium are presented in Table 2, where it can be observed that a good correlation coefficient was obtained with the linear regression model (R^2^ = 0.9952–0.9985), which suggests an adequate fit of the data with the model. The IC_50_ values were 1.74–2.00 mg/mL, while the MIC values were 0.0519–0.121 mg/mL. The lowest MIC and IC_50_ values were obtained for *S.* Enteritidis (0.0519 and 1.74 mg/mL, respectively). This indicates a greater sensitivity of this bacteria to NSps-βCDSL-ACGs. In contrast, the highest values were presented by the *S.* Typhimurium strain (0.121 and 2.00 mg/mL, respectively). The prevalence of *S.* Typhimurium has been reported by analyzing the multiple antibiotic resistance index (MAR) in strains isolated from chickens resistant to them, finding higher resistance in *S.* Typhimurium strains (MAR 0.40) than *S.* Enteritidis strains, which showed less resistance (MAR 0.27). The high resistance of *S.* Typhimurium may be due to a greater exposure of this strain to antibiotics, which has allowed it to generate resistance mechanisms [39].

### 3.4. Lethality of NSps-βCDSL-ACGs Against S. Enteritidis, S. Typhimurium, S. Infantis, and S. gallolyticus

According to the results presented in Table 3, all concentrations of NSps-βCDSL-ACGs decreased the colony count (*p* < 0.05) in all the bacteria evaluated. The lowest logarithmic reduction was observed in the strains of *S.* Infantis (0.16–0.98 log CFU/m), where statistical differences (*p* < 0.05) were observed depending on the concentration of ACGs in each NSps. Likewise, the most substantial reduction was observed with NSps-βCDSL-ACGs at 1.5 and 2.5 mg/mL (1.10–3.19 log CFU/mL) on *S. gallolyticus, S.* Typhimurium, and *S.* Enteritidis strains compared to NSps-βCDSL-ACGs at 0.5 mg/mL (0.65–1.88 CFU/mL., βCDSL complex showed no log reduction, while the positive control showed a low log reduction (1.08–1.42 log CFU/mL) compared to the highest doses of NSps-βCDSL-ACGs.

The results obtained were superior to those reported by Aguilar-Hernández et al. [12] using isolated ACGs, where a 1.81 log CFU/mL reduction was achieved against strains of *E. faecalis*, *L. monocytogenes,* and *Escherichia coli*. The most significant logarithmic reduction obtained in this work can be attributed to the nanometric size (207–239 nm) of the particles in the NSps-βCDSL-ACGs. Nanosuspension properties such as particle size and carrier effect are vital factors that improve the antibacterial activity of active ingredients because they could enter intracellularly and have a better biodistribution. In addition, NSps allow a higher load of compounds and, therefore, greater cellular internalization added to the surface charge and the Z potential of the nanosuspensions that drive interactions with specific proteins in bacteria, compromising bacterial structure and functionality and decreasing viability [40].

## 4. Conclusions

The developed NSps as carriers of acetogenins had nanometer-sized particles, low PDI values, and good stability. In addition, the spectroscopic analysis confirmed the presence of ACGs in NSps. NSps-βCDSL-ACGs at 2.5 mg/mL demonstrated higher bacterial inhibition against *S.* Enteritidis, *S.* Typhimurium, *S.* Infantis, and *S. gallolyticus* at up to 48 h of exposure than positive control. A reduction of up to 3 log CFU/mL was achieved for *S.* Typhimurium. Therefore, NSps-βCDSL-ACGs could be a promising alternative to antibiotics in highly prevalent bacteria in the poultry industry.

## 5. Perspectives

The production of this type of carrier at the industrial level has important possibilities since the stabilizers used in this work are of commercial quality, low cost, and are used in low quantities. On the other hand, agitation as a dispersion method in preparing the nanosuspensions is a relatively simple process that can be easily scaled up. Obtaining the isolated ACG could be the most expensive part. Still, there is a cost benefit due to their effect against highly resistant bacteria, such as those treated in this study.

## Figures and Tables

**Table 1:** Particle size, polydispersity index (PDI), and Z-potential of NSps-βCDSL-ACGs with different concentrations of the active ingredient.

Parameter	NSps-βCDSL-ACGs		
	1 mg/mL	3 mg/mL	5 mg/mL
Particle size (nm)	207.34 ± 7.63 ^c^	228.18 ± 10.05 ^b^	239.12 ± 3.82 ^a^
PDI	0.178 ± 0.024 ^b^	0.136 ± 0.021 ^a^	0.150 ± 0.029 ^ab^
Z potential (mV)	−50.36 ± 0.90 ^a^	−49.02 ± 1.01 ^b^	−47.18 ± 0.58 ^c^

Data are expressed as mean ± SD (n = 3). Different lowercase letters indicate significant statistical differences between treatments (*p* < 0.05). Particle size was obtained from the mean of 20 measurements in triplicate and expressed in nm.

**Table 2:** Determination of the mean inhibitory concentration (IC_50_) and minimal inhibitory concentration (MIC) of the NSps-βCDSL-ACGs through a linear regression model.

Bacteria	IC_50_ (mg/mL)	MIC (mg/mL)	R^2^
*Salmonella* Enteritidis	1.74	0.0919	0.9985
*Salmonella* Typhimurium	2.00	0.0519	0.9980
*Salmonella* Infantis	1.98	0.053	0.9952
*Streptococcus gallolyticus*	1.92	0.121	0.9983

R^2^ corresponds to the correlation coefficient.

**Table 3:** Lethality (Log CFU/mL) of NSps-βCDSL-ACGs (with different concentrations of the active ingredient) against *S.* Enteritidis, *S.* Typhimurium, *S.* Infantis, and *S. gallolyticus* after 48 h of exposure.

Bacteria	* Positive Control	NSps-βCDSL-ACGs		
		0.5 mg/mL	1.5 mg/ mL	2.5 mg/mL
*S.* Enteritidis	1.08 ± 0.03 ^c^	1.07 ± 0.47 ^b^	1.70 ± 0.13 ^a^	1.54 ± 0.15 ^a^
*S.* Typhimurium	1.29 ± 0.35 ^c^	1.88 ± 0.23 ^b^	3.03 ± 0.32 ^a^	3.19 ± 0.10 ^a^
*S.* Infantis	0.73 ± 0.63 ^ab^	0.16 ± 0.03 ^b^	0.25 ± 0.60 ^ab^	0.98 ± 0.30 ^a^
*S. gallolyticus*	1.42 ± 0.39 ^a^	0.65 ± 0.58 ^ab^	1.10 ± 0.25 ^ab^	1.16 ± 0.15 ^b^

* Positive control = ampicillin for *S.* Enteritidis (2.5 μg/mL), *S.* Typhimurium (0.75 μg/mL), *S.* Infantis (2 μg/mL), and *S. gallolyticus* (0.5 μg/mL). Data are expressed as mean ± SD. Different lowercase letters indicate significant statistical differences between treatments (*p* < 0.05).

## Data Availability

Data will be made available upon request.
